# A novel integrated *in vitro* method for evaluating the moisturizing performance of injectable sodium hyaluronate

**DOI:** 10.1093/rb/rbag013

**Published:** 2026-02-09

**Authors:** Jianfeng Shi, Wenna Xu, Hongfu Liu, Fengjuan Shan, Rui Wang, Linnan Ke, Qianqian Han, Yong Lu

**Affiliations:** Institute for Medical Devices Control, National Institutes for Food and Drug Control, Beijing 102629, China; National Institutes for Food and Drug Control, Beijing 102629, China; School of Traditional Chinese Pharmacy, China Pharmaceutical University, Nanjing, Jiangsu 210009, China; Shandong Engineering Research Center for Tissue Engineering and Regenerative Medicine, Jinan, Shandong 250100, China; Department of Anatomy, Binzhou Medical University, Yantai, Shandong 264003, China; Shandong Engineering Research Center for Tissue Engineering and Regenerative Medicine, Jinan, Shandong 250100, China; Institute for Medical Devices Control, National Institutes for Food and Drug Control, Beijing 102629, China; National Institutes for Food and Drug Control, Beijing 102629, China; Institute for Medical Devices Control, National Institutes for Food and Drug Control, Beijing 102629, China; National Institutes for Food and Drug Control, Beijing 102629, China; Institute for Medical Devices Control, National Institutes for Food and Drug Control, Beijing 102629, China; National Institutes for Food and Drug Control, Beijing 102629, China; National Institutes for Food and Drug Control, Beijing 102629, China

**Keywords:** injectable sodium hyaluronate, moisturizing performance, *in vitro* evaluation, HaCaT cells, reconstructed human full-thickness skin, CD44, AQP3, natural moisturizing factors

## Abstract

Injectable sodium hyaluronate (NaHA) is extensively utilized in aesthetic medicine as a dermal hydrating agent. However, there are few standardized, human-relevant preclinical methods that can reliably evaluate the moisturizing performance of those products. Existing animal and simplified cell-based models show restricted physiological relevance and insufficient sensitivity. To address this gap, we established a depot-mimicking reconstructed human full-thickness skin (RhFS) platform incorporating an inclusion-based intradermal delivery strategy. Instead of conventional mixing, this strategy mimics the clinical ‘depot effect’ of intradermal injection, allowing for the simultaneous assessment of cellular responses and tissue-level hydration dynamics. It forms a localized NaHA depot within the dermal compartment of RhFS and preserves spatial hydration gradients, which are lost when NaHA is mixed homogenously. The platform integrates physicochemical characterization of polymer-bound water states with cellular and tissue-level functional readouts. By quantifying key biomarkers, including CD44, aquaporin-3 (AQP3) and natural moisturizing factors (NMFs), our results demonstrate that the inclusion-based delivery strategy significantly outperforms conventional mixing approaches in activating epidermal hydration pathways. Crucially, this platform effectively distinguished the moisturizing efficacy of multiple commercial NaHA formulas, thereby revealing a structure-activity relationship between water-binding states and biological outcomes. Overall, this study presents a reproducible, mechanism-informed and human-relevant framework for preclinical performance evaluation of NaHA-based injectable biomaterials and provides a sensitive alternative to conventional animal-based approaches.

## Graphical abstract

**Figure:**
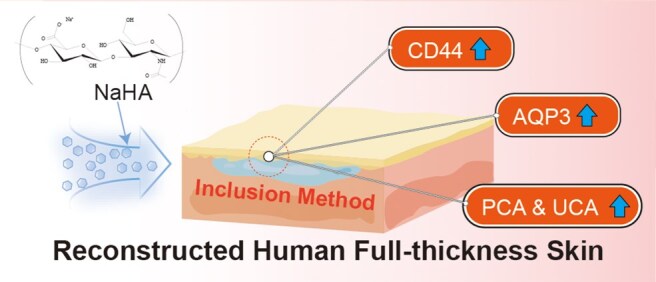

## Introduction

Hyaluronic acid (HA), a naturally occurring glycosaminoglycan known for its exceptional water-binding capacity [1], has become a foundational component of modern aesthetic medicine. While crosslinked HA is traditionally employed for volumetric augmentation, there is a growing paradigm shift toward using un-crosslinked or lightly crosslinked sodium hyaluronate (NaHA) solutions for skin rejuvenation. When injected into the superficial dermis, these formulations—often termed ‘skin boosters’—aim to restore the extracellular matrix (ECM) [2, 3], enhance hydration and improve skin elasticity [4]. Driven by surging consumer demand, the market for such injectable hydrating products has expanded rapidly [5]. However, this clinical proliferation has outpaced the development of standardized preclinical evaluation systems, creating a ‘validation gap’ that complicates regulatory approval and product quality control.

Establishing robust efficacy evidence for these products remains a significant challenge. Historically, preclinical evaluation has relied heavily on animal models. However, these models often fail to predict human outcomes due to fundamental anatomical discrepancies; for instance, the lack of rete ridges in rodent skin impedes the accurate simulation of human transdermal nutrient transport [6]. Furthermore, a critical but often overlooked limitation is the rheological mismatch: the low viscosity of un-crosslinked NaHA solutions leads to rapid diffusion and insufficient tissue retention in small animal models, making it difficult to assess local moisturizing effects accurately. Although large-animal models like pigs offer better structural resemblance, their high cost and inherent variability render them impractical for high-throughput screening or routine quality control [7, 8]. Concurrently, the global regulatory shift toward the 3R principles (replacement, reduction and refinement) has intensified the urgency for human-relevant *in vitro* alternatives [9, 10].

In response to these limitations, *in vitro* systems utilizing human immortalized keratinocytes (HaCaT) and reconstructed human full-thickness skin (RhFS) have emerged as ethical and physiologically relevant platforms [11–13]. These models allow for the precise interrogation of hydration mechanisms, such as the regulation of aquaporin-3 (AQP3) [14, 15], CD44 receptors, and natural moisturizing factors (NMFs) [16–18]. However, most existing *in vitro* protocols are designed for topical cosmetics and fail to replicate the unique biomechanical and concentration gradients created by intradermal injection.

To bridge this translational gap, this study establishes an integrated *in vitro* evaluation framework tailored specifically for injectable NaHA. We combined HaCaT cell-based assays with an RhFS model to construct a hierarchical assessment system spanning from cellular signaling to tissue-level functionality. A key innovation of this work is the establishment of a depot-mimicking RhFS evaluation platform based on an inclusion-based intradermal delivery strategy. Unlike conventional homogeneous exposure paradigms, this platform enables localized NaHA retention within the dermal compartment, thereby reconstructing a clinically relevant hydration depot and the associated dermal–epidermal hydration gradient. Importantly, this platform is designed not only to assess biological efficacy, but also to functionally link the physicochemical water-binding states of NaHA with epidermal hydration outcomes, using CD44, AQP3, and NMFs as mechanism-informed biomarkers. By validating this platform against commercial products, we aim to provide a standardized, mechanistically informative and regulatory-compliant tool for the efficacy assessment of injectable hydrating biomaterials.

## Materials and methods

### Reagents and assay kits

HuFullKutis Full-Thickness Skin Model and model culture medium were supplied by Jinan Pansheng Biotechnology Co., Ltd. HaCaT cells were purchased from National Collection of Authenticated Cell Cultures. Dulbecco’s Modified Eagle Medium (DMEM) and fetal bovine serum (FBS) were purchased from Gibco. Phosphate buffered saline (PBS) and Trypsin-EDTA were purchased from HyClone. Ammonium formate and methanol were obtained from Macklin. Acetonitrile was purchased from Concord. Proteinase K, penicillin-streptomycin solution (PS), TdT-mediated dUTP Nick-End Labeling (TUNEL) Apoptosis Assay Kit (Green Fluorescence), Reactive Oxygen Species (ROS) Assay Kit (Green Fluorescence), Cell Counting Kit-8 (CCK-8) kit and 4% paraformaldehyde were purchased from Solaibio. Deionized water was purchased from Grade I. Absolute ethanol was purchased from Sinopharm. Eco-friendly dewaxing transparent solution, citrate antigen repair solution (pH 6.0), EDTA antigen repair solution (pH 9.0), tissue autofluorescence quencher, bovine serum albumin (BSA), Anti-AQP3 Rabbit pAb, Anti-CD44 Rabbit pAb, Cy3-conjugated goat anti-rabbit IgG, DAPI staining reagent and anti-fade mounting medium were purchased from Servicebio. RNeasy Mini Kit was purchased from QIAGEN. One Step TB Green PrimeScript RT PCR Kit was purchased from TAKARA.

### Sample collection

Four types of injectable NaHA products were collected from the Chinese market and numbered sequentially (e.g. A–D, concentrations 10, 15, 15 and 5 mg/mL, respectively).

### Fourier-transform infrared (FTIR) spectroscopy

The structural characteristics of samples A–D were analyzed using FTIR spectroscopy (Thermo Nicolet iS50, USA). Before measurement, each sample was lyophilized in a desiccator for 4 h to eliminate residual moisture. Approximately 1–2 mg of the dried sample was finely ground with potassium bromide (KBr) and pressed into pellets. The spectra were recorded over the range of 4000–500 cm^−1^ with a resolution of 4 cm^−1^. Each spectrum was averaged over 32 scans to enhance the signal-to-noise ratio.

### Raman spectroscopy analysis

Raman spectra were acquired to characterize the molecular structure and hydration states of the NaHA samples using a confocal Raman microspectrometer (LabRAM Soleil, HORIBA) equipped with a 532 nm laser. The acquisition parameters were set as follows: a 600 grooves/mm grating, a 50× objective lens and an acquisition time of 10 s.

For the analysis of liquid NaHA samples, the laser power was maintained at 67 mW; however, to enhance the signal-to-noise ratio for the characteristic spectral regions of NaHA, the power was increased to 84 mW and the acquisition time was extended to 30 s.

To investigate the water-binding capacity, the samples were lyophilized to remove free water. NaHA solutions were dispensed into 35 mm dishes and subjected to a two-stage freeze-drying process: primary drying at −40°C for 2 h, followed by secondary drying at −80°C for 2 h. Spectra of the resulting lyophilized powders were collected under identical optical conditions. Data processing, including baseline correction for fluorescence removal and normalization to the 3100–3700 cm^−1^ region, was performed using LabSpec 6 software. The O–H stretching band was subsequently deconvoluted to quantify different water-binding states.

### Molecular weight and distribution analysis

The molecular weight and polydispersity of samples A–D were determined using high-performance size-exclusion chromatography coupled with multi-angle laser light scattering (HPSEC-MALLS). The system comprised an Agilent 1260 high-performance liquid chromatography (HPLC) equipped with UV and refractive index detectors, connected to a Wyatt DAWN HELEOS II multi-angle light scattering detector (18 angles). The mobile phase consisted of a 0.2 mol/l NaCl solution, prepared by dissolving 11.7 g of NaCl in water to a final volume of 1000 mL, followed by filtration through a 0.22 μm membrane and degassing. Sample solutions were created by dissolving approximately 1 mg/mL of NaHA in the mobile phase, which was also filtered through a 0.22 μm membrane filter. Chromatographic separation utilized a Shodex SB-806HQ gel permeation column (8.0 mm × 300 mm) at a flow rate of 1.0 mL/min, with an injection volume of 100 μL. The column was equilibrated until a stable baseline was established prior to analysis. Data acquisition and analysis were performed using ASTRA software (Wyatt Technology). The refractive index increment (d*n*/d*c*) was set at 0.160 mL/g, and the weight-average molecular weight (Mw), number-average molecular weight (Mn) and polydispersity index (Mw/Mn) were calculated according to the software’s standard protocol.

### Rheological analysis

The rheological properties of samples A–D were assessed using a Physica MCR301 rheometer (Anton Paar, Austria) with a cone–plate geometry (CP50-1, diameter 50 mm, cone angle 1°). The plate-to-plate gap was maintained at 0.110 mm. Viscosity measurements were performed in flow sweep mode at a controlled temperature of 25 ± 0.2°C. The shear rate was varied logarithmically from 0.001 to 1000 s^−1^, and the corresponding apparent viscosity was recorded. For comparative analysis, representative viscosity values were extracted at shear rates of 0.0562, 0.1, 0.562, 1, 5.62, 10, 56.2 and 100 s^−1^ for comparative analysis.

### Cell culture

HaCaT cells were grown in DMEM supplemented with 10% FBS and 1% PS (complete culture medium). Cells were passaged every 2 days to ensure optimal growth conditions. Full-thickness skin model was subjected to submerged in model culture medium. Full-thickness skin model and cells were maintained at 37°C with a 5% CO_2_ atmosphere.

### Cell viability determination

The cytotoxicity and proliferative effects of NaHA were evaluated using the CCK-8 assay. HaCaT cells were cultured in a 96-well plate at a density of 1 × 10^5^ cells per well and incubated for 24 h. The culture medium was then replaced with serial dilutions of Sample D prepared in complete culture medium, with final volume ratios ranging from 6.125% to 100%. After a 24-h incubation, 10% (v/v) CCK-8 solution was added to each well, and the cells were incubated for an additional 2 h. The optical density (OD) was measured at 450 nm using a microplate reader (M5, Molecular Devices).

### Scratch wound assay

To assess cell migration, HaCaT cells were seeded at 5 × 10^5^ cells/well in a 6-well plate and cultured until reaching 90% confluence. A linear scratch was created across the cell monolayer using a sterile 200μL pipette tip. Following two washes with PBS to remove cellular debris, the cells were incubated in serum-free DMEM containing Sample D at volume ratios of 25%, 50% or 75%. Serum-free DMEM alone was used as the negative control. Images of the wound area were captured at 0, 12 and 24 h using an inverted fluorescence microscope (Ts2R-FL, Nikon), and the wound closure rate was quantified using ImageJ software.

### Cell drying damage assay

HaCaT cells were cultured in a 96-well plate at a density of 1 × 10^5^ cells per well and incubated for 24 h. Following this incubation, each well was washed twice with PBS. The cells were then treated for 24 h with solutions prepared in complete culture medium: Sample D was diluted to final volume ratios of 25%, 50%, and 75%; PBS served as the model control and 5% (v/v) glycerol was used as the positive control. Cells treated with complete culture medium alone functioned as the untreated blank control, representing a non-damaged baseline. After 24 h, the liquid was completely aspirated from each well. The 96-well plate was placed in a biosafety cabinet and subjected to drying damage treatment for 20 min at room temperature with an air velocity of 0.32–0.4 m/s. The cells in the blank control wells were not aspirated and did not undergo drying stress. The CCK-8 kit was employed to assay cell viability according to the previously described method. The cell Meter TUNEL Apoptosis and ROS Assay Kit were utilized to characterize cellular damage, and the stained cells were examined using a confocal laser scanning microscope (FV4000, Olympus) with 488 nm excitation light for green fluorescence. The mean fluorescence intensity (MFI) was analyzed using ImageJ software.

### *In vitro* gene expression assay

The mRNA expression levels of hydration-related genes (*AQP3*, *CD44*, and *FLG*) were quantified using quantitative real-time PCR (qRT-PCR). Total RNA was extracted from HaCaT cells using the RNeasy Mini Kit following the manufacturer’s protocol. Reverse transcription and amplification were performed using the One Step TB Green PrimeScript RT-PCR Kit on a Real-Time PCR Detection System (QuantStudio5, Bio-Rad). Relative gene expression was calculated using the 2^−△△^^*Ct*^ method, with β-actin serving as the internal reference. The primer sequences used are listed in Table 1.

**Table 1:** Primer sequences.

Genes	Forward primer (5′–3′)	Reverse primer (5′–3′)
β-Actin	CTCCATCCTGGCCTCGCTGT	GCTGTCACCTTCACCGTTCC
AQP3	GCTCCATTGCGGGTGTCTTCGTGTA	TGGACAGTCAGTGGATGCTCAAGGC
CD44	CATCTACCCCAGCAACCCTA	ACTGTCTTCGTCTGGGATGG
FLG	TGAAGCCTATGACACCACTGA	TCCCCTACGCTTTCTTGTCCT

### Construction of RhFS containing sodium hyaluronate

RhFS models were engineered using two distinct NaHA delivery strategies: the mixing method and the inclusion method.

For the inclusion method (designed to mimic intradermal depots), a collagen layer was first prepared by mixing dermal fibroblasts, culture medium and collagen solution. This mixture was added to a Transwell insert. Prior to complete gelation, a standardized indentation (approximate depth: 2.1 mm) was created on the collagen surface using a sterile glass ring, guided by a reference mark on the insert wall to ensure consistent depth. Sample D (100 μL) was dispensed specifically into this indentation, and the remaining collagen–fibroblast mixture was layered on top to encapsulate the NaHA depot.

For the mixing method, 100 μL of Sample D was directly homogenized with the dermal fibroblast–collagen mixture before being cast into the Transwell insert, resulting in a dispersed distribution of NaHA throughout the dermal equivalent.

RhFS models without NaHA served as controls. All constructs were cultured for 7 days to allow dermal maturation. Subsequently, an epidermal cell suspension was seeded onto the dermal surface and cultured under submerged conditions for 4 days. Finally, the models were lifted to the air-liquid interface and cultured for 10 days to promote epidermal stratification and cornification.

### Extraction of natural moisturizing factors

The RhFS was carefully excised along the edge of the Transwell insert using a blade. The epidermal layer was then separated with forceps and transferred to a 1.5 mL microcentrifuge tube. Subsequently, 500 μL of proteinase K working solution (0.2 mg/mL) was added, and the samples were incubated at 50 °C for 1 h to enzymatically digest the non-stratum corneum (SC) components. The isolated SC was collected with forceps and transferred to a new 1.5 mL tube, followed by the addition of 250 μL methanol. The samples were sonicated for 30 min and then centrifuged at 14 000 rpm at 4°C for 10 min. The supernatant was transferred to a new tube and evaporated to dryness using a nitrogen evaporator. The residue was reconstituted in 250 μL of ultrapure water and sonicated for an additional 30 min. After centrifugation at 12 000 rpm at 4°C for 10 min, 194 μL of the resulting supernatant was transferred to an HPLC vial insert. Finally, 5 μL of a 1 mol/L ammonium formate stock solution and 2.5 μL of acetonitrile were added. The mixture was vortexed thoroughly to yield the final sample solution for analysis.

### Confocal Raman imaging

The PBS and NaHA were delivered into RhFS by the inclusion method, and the normally cultured RhFS served as control. Confocal Raman imaging (LabRAM Soleil, HORIBA) was performed to assess hydration-related spectral changes within the epidermal–dermal interface following inclusion-based delivery. Measurements were conducted within the superficial region of the model (approximately 200 μm from the surface) to monitor time-dependent changes in tissue water content. Raman maps were measured by LabSpec 6 software.

### Quantification of natural moisturizing factors

The sample solution was analyzed using HPLC (LC-20A, SHIMADZU) under the following conditions, a C18 column (5 μm, 4.6 × 250 mm); a mobile phase consisting of 990 mL of 20 mmol/L ammonium formate solution mixed with 10 mL of acetonitrile; a flow rate of 0.4 mL/min; column temperature of 30°C and an injection volume of 40 μl. Detection wavelengths were set at 270 nm for urocanic acid (UCA) and 210 nm for pyrrolidone carboxylic acid (PCA). The retention times were 7.2 min for PCA, 9.9 min for trans-UCA and 17.9 min for cis-UCA.

### Immunofluorescent staining assay

At predetermined time points, RhFS were excised as previously described, and frozen tissue sections were prepared for immunofluorescence staining. The sections were incubated overnight at 4°C with a primary antibody (anti-CD44/AQP3, rabbit polyclonal antibody). After washing, the sections were incubated for 50 min at room temperature in the dark with a Cy3-conjugated goat anti-rabbit IgG secondary antibody. Subsequently, DAPI staining solution was applied to counterstain the nuclei, followed by a 10-min incubation at room temperature in the dark. Fluorescence images were acquired using a confocal laser scanning microscope (D-Eclipse C1, Nikon). Semiquantitative analysis of MFI was conducted using ImageJ software.

### Global and differential gene expression analysis

HaCaT cells were categorized into four groups. The experimental group received gradient solutions of Sample D at volume ratios of 10%, 25%, and 50% for 24 h. In contrast, the negative control group was cultured under the same conditions without NaHA supplementation. After treatment, total RNA was extracted and subjected to high-throughput sequencing. Raw sequencing reads underwent quality checks and normalization prior to analysis. The RNA sequencing data generated in this study have been submitted to the China National Center Bioinformation and are currently under administrative review. The accession number will be provided upon approval and public release of the dataset. Differential expression analysis was conducted using the DESeq2 package, with thresholds established at a fold change greater than 1.5 and an adjusted *P*-values of less than 0.05. Genes that met these criteria were classified as differentially expressed genes (DEGs). Functional annotation and pathway enrichment analyses of DEGs were performed using the Kyoto Encyclopedia of Genes and Genomes (KEGG) database. Pathways with enrichment *P*-values less than 0.05 were deemed significantly associated with the treatment response.

### Statistical analysis

All data were expressed as mean ± standard deviation (SD). For comparisons among multiple dose groups, one-way ANOVA followed by Tukey’s *post hoc* test was applied. For two-group comparisons between delivery methods, unpaired two-tailed Student’s *t*-test was used. A *P*-value <0.05 was considered statistically significant. Statistical analyses and data visualization were performed using GraphPad Prism 9.5.

## Results

### Physicochemical characterization of NaHA

**Figure 1:**
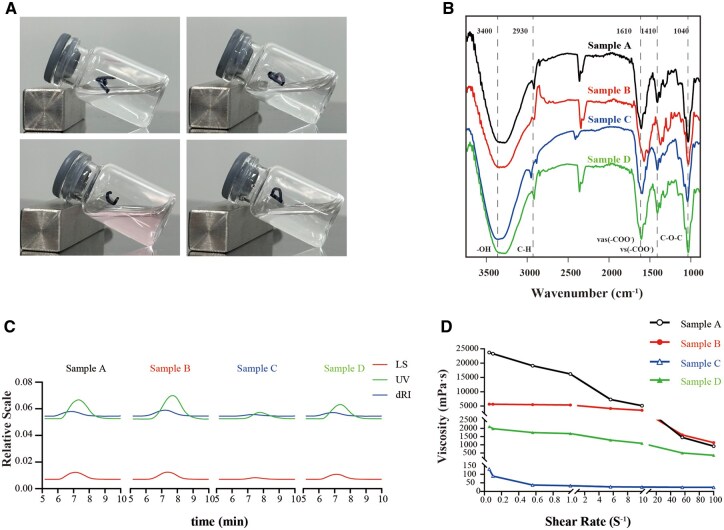
Physicochemical characterization of injectable NaHA solutions (Samples A–D). (**A**) Macroscopic observation of Samples A–D. (**B**) FTIR spectra of lyophilized NaHA samples display characteristic absorption bands: ∼3400 cm^−1^ (–OH stretching), ∼2930 cm^−1^ (C–H stretching), ∼1610 cm^−1^ and ∼1410 cm^−1^ (asymmetric and symmetric stretching of –COO^−^ groups), and ∼1040 cm^−1^ (C–O–C stretching of glycosidic bonds). (**C**) Molecular weight distribution of NaHA samples was determined using multi-angle light scattering coupled with HPLC. All samples exhibited unimodal profiles without aggregation peaks. (**D**) Rheological properties of NaHA solutions demonstrated shear-thinning behavior across shear rates ranging from 0.001 to 1000 s^−1^. The viscosity was correlated with both molecular weight and concentration. Sample C (Mw 4.475 × 10^6^ Da, 15 mg/ml) exhibited the lowest viscosity, while Sample A (Mw 1.35 × 10^6^ Da, 10 mg/mL) displayed the highest viscosity.

These four solutions are all transparent liquids exhibiting a certain level of viscosity (Figure 1A). Except for sample C, which is light pink, all other samples are colorless.

FTIR analysis confirmed the structural integrity of all four samples (A–D) (Figure 1B). The spectra exhibited characteristic absorption bands for NaHA: a broad band at approximately 3400 cm^−1^ corresponding to the –OH stretch, a peak at around 2930 cm^−1^ for the C–H stretch, and distinct vibrations of the carboxylate group approximately at 1610 cm^−1^ (asymmetric –COO^−^ stretch) and 1410 cm^−1^ (symmetric –COO^−^ stretch). Additionally, the presence of a band near 1040 cm^−1^, corresponding to C–O–C stretching of glycosidic bonds further substantiated the polysaccharide structure. These features were consistent across all samples, thereby confirming their chemical identity.

Molecular weight analysis using gel permeation chromatography demonstrated a unimodal distribution profile for all four samples. This finding confirms structural homogeneity and indicates the absence of apparent aggregation (Figure 1C). The calculated Mw and the Mw/Mn are summarized in Table 2.

**Table 2:** Mw and Mw/Mn.

Sample	Mw	Mw/Mn
A	1.353 × 10^6^ (±3.595%)	1.701 (±10.287%)
B	7.372 × 10^5^ (±3.470%)	1.125 (±6.994%)
C	4.475 × 10^5^ (±3.119%)	1.068 (±4.718%)
D	1.095 × 10^6^ (±3.876%)	1.247 (±13.790%)

All four NaHA solutions exhibited pronounced shear-thinning behavior, characterized by a rapid decrease in apparent viscosity as the shear rate increased (Figure 1D). This non-Newtonian property is typical of entangled polymer networks and is essential for facilitating injectability. At comparable concentrations, the viscosity profiles were correlated with Mw. Sample C, which has the lowest Mw (4.475 × 10^5^ Da, 15 mg/mL), displayed the lowest viscosity across the tested shear rates, indicating a weaker entangled network. Conversely, Sample A, with the highest Mw (1.35 × 10^6^ Da, 10 mg/mL) demonstrated the highest viscosity, reflecting dense chain entanglement despite comparable concentration. Samples B (7.37 × 10^5^ Da, 15 mg/mL) and D (1.09 × 10^6^ Da, 5 mg/mL) exhibited intermediate viscosities, with the lower concentration of Sample D mitigating its moderate Mw.

Collectively, these analyses confirmed that all NaHA formulations preserved structural integrity while exhibiting distinct molecular weight distributions and rheological behaviors.

### Water-binding capacity analysis

**Figure 2:**
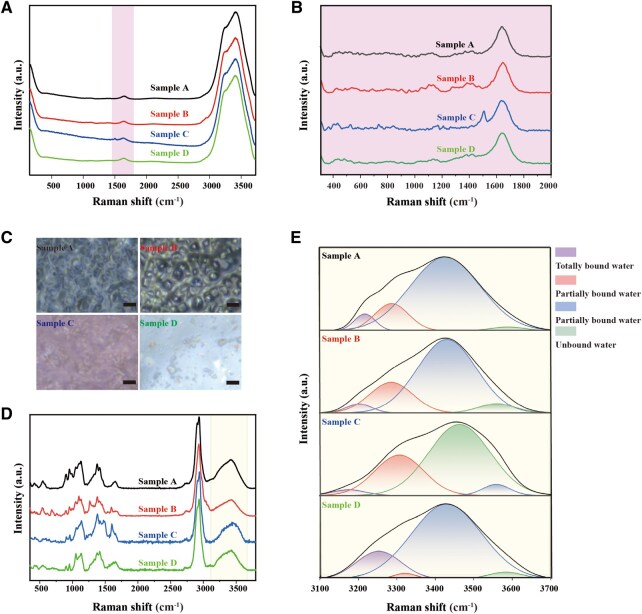
Water-binding capacity analysis of Samples A–D. (**A**) Raman spectra of Samples A–D. (**B**) Raman spectra in the fingerprint region. (**C**) Morphology of the lyophilized samples observed via optical microscopy, with a scale bar of 5 μm. (**D**) Raman spectra of the four samples following lyophilization. (**E**) Curve fitting of the –OH band from the lyophilized samples.

To investigate the hydration mechanism at the molecular level, we utilized Raman spectroscopy to characterize the water-binding states within the NaHA matrix. Initial spectra of the liquid samples revealed a dominant hydroxyl stretching band in the 3100–3700 cm^−1^ region, masking specific polymer–water interactions (Figure 2A). We then performed a long-term analysis of the characteristic peak areas of the samples. Although the overall signals were weak, a measurable signal was detected, with a characteristic peak at 1500 cm^−1^ observed exclusively in Sample C (Figure 2B). To isolate the signals of bound water, samples were lyophilized. Morphological observation indicated that while Samples A and B formed dense, continuous structures, Samples C and D yielded thinner, more porous films, likely attributable to differences in molecular weight and concentration (Figure 2C). Following a previous study [19], deconvolution analysis of the O–H stretching band (Figure 2D and E) in the lyophilized samples identified four distinct water species: totally bound (3210 cm^−1^), partially bound A (3280 cm^−1^), partially bound B (3345 cm^−1^) and unbound water (3470 cm^−1^).

Quantitative analysis of the integrated peak areas revealed significant variations in hydration profiles among the formulations (Table 3). Notably, Sample D exhibited the highest proportion of totally bound water (16.6%), markedly superior to Samples A (5.3%), B (3.7%) and C (2.6%). This unique ability to tightly sequester water molecules suggests a superior potential for sustained hydration in tissue.

**Table 3:** Analysis of various types of bound water.

Sample	Ratio of bound water (%)				Ratio of unbound water (%)
	Totally bound	Partially bound A	Partially bound B	Subtotal (%)	
A	5.3	14.0	78.8	98.1	1.9
B	3.7	21.2	69.7	94.6	5.4
C	2.6	29.0	4.4	36.0	64.0
D	16.6	1.3	81.6	99.5	0.5

Among the four formulations tested, Sample D exhibited the superior water-binding capacity. Consequently, it was selected as the representative formulation for subsequent mechanistic and tissue-level evaluations, rather than for comparative performance ranking.

### Effects of NaHA on HaCaT cell viability and migration

**Figure 3:**
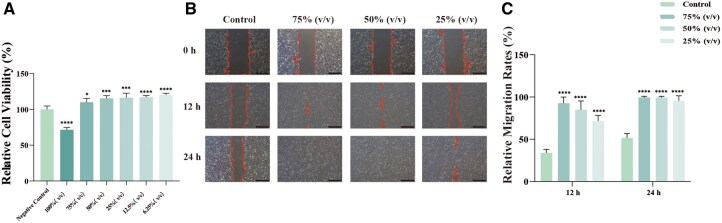
NaHA enhances the proliferation and migration of HaCaT cells. (**A**) Relative cell viability was assessed after a 24-h exposure to various concentrations of sample (*n* = 4). *P < 0.05, ***P < 0.001, ****P < 0.0001, compared to the negative control. (**B**) Images from the scratch wound healing assay were captured at 12 and 24 h post-treatment. Scale bar = 500 μm. (**C**) Quantitative analysis of the wound healing percentage is presented (*n* = 3). *P < 0.05, ****P < 0.0001, compared to the control.

To evaluate the biological effects of NaHA at the cellular level, we first assessed its impact on the viability and migration of HaCaT cells. The CCK-8 assay demonstrated excellent biocompatibility across a wide concentration range (Figure 3A). Even in its pure form, without serum or medium, relative cell viability remained above 70%, indicating acceptable cytotoxicity according to ISO 10993-5 standards. Notably, viability in the 75% (v/v) sample treatment group exceeded that of the control, leading to its selection as the optimal dose for subsequent experiments.

Consistent with these results, the scratch wound assay revealed that sample treatment significantly enhanced cell migration. The wound area was markedly reduced in the NaHA-treated groups compared to the control at both 12 and 24 h post-scratch (Figure 3B and C). Collectively, these findings suggest that NaHA not only exhibits high biocompatibility but also promotes the proliferation and motility of HaCaT cells.

### Protective effects of NaHA against desiccation-induced cellular damage

**Figure 4:**
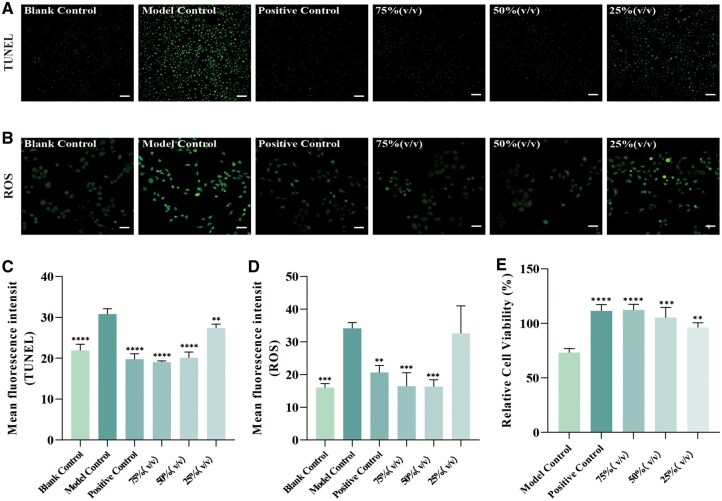
NaHA protects HaCaT cells from drying-induced damage. (**A**) Immunofluorescence staining of TUNEL, scale bar = 100 μm. (**B**) Immunofluorescence staining of ROS, scale bar = 50 μm. (**C, D**) The fluorescence intensity of TUNEL (*n* = 4) and ROS (*n* = 3). (**E**) The relative cell viability (*n* = 6). **P < 0.01, ***P < 0.001, ****P < 0.0001, compared to the model control.

We established an *in vitro* air-drying model to evaluate the cytoprotective capacity of NaHA under dehydration stress. Desiccation treatment induced severe cellular damage in HaCaT cells, characterized by a significant elevation in apoptosis (indicated by TUNEL staining) and oxidative stress (indicated by ROS fluorescence) compared to the blank control (Figure 4A and B). Pretreatment with NaHA effectively mitigated these stress responses in a dose-dependent manner. Cells treated with gradient concentrations of sample showed significantly reduced MFI for both TUNEL and ROS, indicating the preservation of genomic integrity and redox homeostasis (Figure 4C and D). Consistent with these findings, the CCK-8 assay confirmed that NaHA treatment rescued cell viability. The 75% (v/v) maintained viability levels comparable to the positive control (glycerol) and significantly higher than the untreated model control (Figure 4E). These results demonstrate that NaHA confers potent cyto-protection against dehydration.

### Transcriptome analysis reveals NaHA-induced upregulation of hydration markers in the epidermis

**Figure 5:**
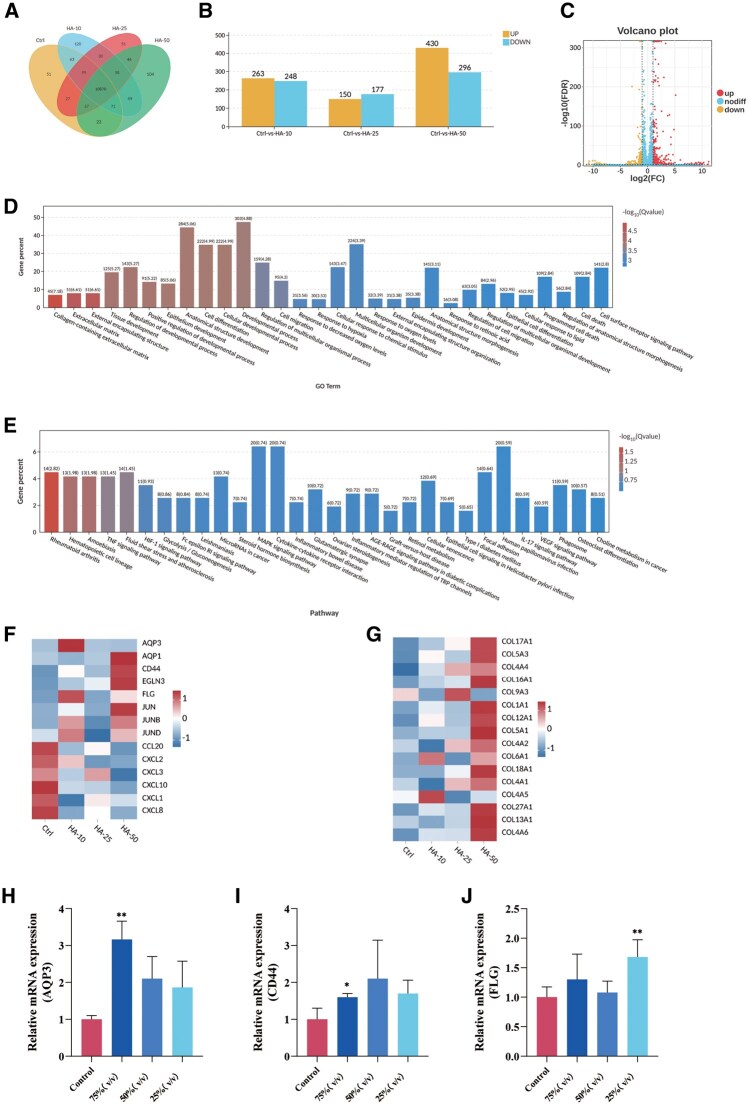
The transcriptome analysis revealed the potential mechanism of NaHA. (**A**) The number of total detected genes and DEGs identified in comparisons between the control group and treatments with 10%, 25% and 50% (v/v) sample. (**B**) The number of upregulated and downregulated DEGs in each comparison group (control vs. 10%, 25% and 50% (v/v) sample). (**C**) Volcano plot of the DEGs between the control and 50% (v/v) sample treatment groups. Genes with significant upregulation and down-regulation are highlighted (log2 fold change > |1| and adjusted *P*-values <0.05). (**D**) GO enrichment analysis of the DEGs, showing significant enrichment in terms related to the ECM and collagen-containing ECM. (**E**) KEGG pathway enrichment analysis of the DEGs. (**F, G**) Heatmaps displaying the expression patterns of key DEGs of interest. NaHA treatment upregulated genes involved in hydration (e.g. *AQP3*, *CD44*, *FLG*), the AP-1 transcription factor family (e.g. *JUN*, *JUNB*, *JUND*), and collagen genes, while down-regulating pro-inflammatory chemokines (e.g. *CCL20*, *CXCL2*); (A–G) (*n* = 3). (H–I) Transcriptional expression of *AQP3*, *CD44* and *FLG* evaluated by RT-PCR (*n* = 6). *P < 0.05, **P < 0.01, compared to the control.

To elucidate the signaling pathways responsible for the moisturizing effects of NaHA, we conducted RNA sequencing analysis. Due to the high viscosity of the 75% (v/v) Sample D treatment, which adversely affected RNA quality, we adjusted the concentrations for sequencing to 10%, 25% and 50% (v/v). RNA sequencing quality control met the acceptance criteria, with all samples passing library preparation and sequencing requirements. A total of 20 376 genes were identified. Differential expression analysis revealed 511, 327 and 726 DEGs for the 10%, 25% and 50% (v/v) treatments compared to the control, respectively (Figure 5A). Among these, 263, 150 and 430 genes were upregulated, while 248, 177 and 296 genes were downregulated, respectively (Figure 5B). A volcano plot depicts the DEG profile for the 25% (v/v) Sample D treatment (Figure 5C).

Gene ontology (GO) and KEGG enrichment analyses were performed to identify the biological processes and pathways affected. The GO analysis demonstrated significant enrichment of DEGs in terms related to the ECM, particularly those associated with collagen-containing structures (Figure 5D). The KEGG pathway analysis revealed enrichment in signaling pathways responsive to inflammation and stress, including *TNF*, *MAPK* and *HIF-1* signaling (Figure 5E). These findings indicate that NaHA enhances epidermal moisturization by reinforcing ECM integrity and attenuating inflammatory and environmental stress responses.

Heatmaps highlight key DEGs of interest (Figure 5F). The expression of the *AQP3* was significantly elevated, along with the upregulation of hydration-related genes *CD44* and *filaggrin* (*FLG*). This finding suggests a potential role for these genes in NaHA-mediated hydration. To validate this transcriptional profile, we performed RT-PCR analysis on *AQP3*, *CD44* and *FLG* in HaCaT cells treated with gradient concentrations of NaHA, which produced consistent results (Figure 5H and I).

Additionally, components of the AP-1 signaling axis, including *JUN*, *JUNB* and *JUND*, were increased, while pro-inflammatory chemokines such as *CCL20* and *CXCL2* were downregulated. This indicates that NaHA mitigates inflammatory signaling. Furthermore, a pronounced upregulation of multiple collagen genes was observed, further supporting the role of NaHA in reinforcing the dermal ECM network (Figure 5G).

### NaHA-induced upregulation of epidermal hydration markers

**Figure 6:**
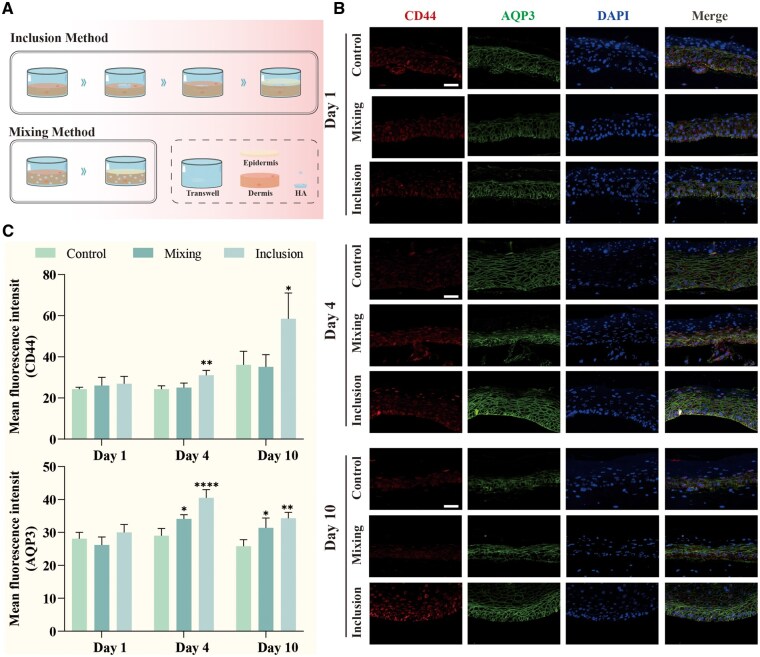
NaHA upregulates the expression of CD44 and AQP3 in RhFS models. (**A**) Schematic illustration of the construction of NaHA-containing RhFS model using mixing method and inclusion method. (**B**) Representative images of CD44 and AQP3 staining in the epidermal layers. Nuclei were counterstained with DAPI, scale bar = 50 μm. (**C**) Quantification of MFI for CD44 and AQP3 (*n* = 4). *P < 0.05, **P < 0.01, ****P < 0.0001, compared to the control.

To determine whether the delivery strategy influences biological efficacy, we compared the expression of CD44 and AQP3 in RhFS models constructed using either the ‘mixing’ or ‘inclusion’ method (Figure 6A). Immunofluorescence analysis was performed on days 1, 4 and 10 of the air–liquid interface culture.

Initially (Day 1), basal expression levels of CD44 and AQP3 were comparable across all groups. However, by Day 4, a distinct divergence in expression profiles emerged. The inclusion method induced the most robust upregulation of both markers, with fluorescence intensities significantly exceeding those of the control and mixing groups. While the mixing method did elevate AQP3 levels relative to the control, it failed to induce a significant increase in CD44 expression. Crucially, the stimulatory effect of the inclusion strategy was sustained through Day 10, whereas the signal in other groups plateaued or declined (Figure 6B and C). These findings suggest that the localized depot formed by the inclusion method provides a more intense and prolonged stimulus to the epidermal hydration machinery than homogeneous dispersion.

### Evaluation of moisturizing capacity based on RhFS

**Figure 7:**
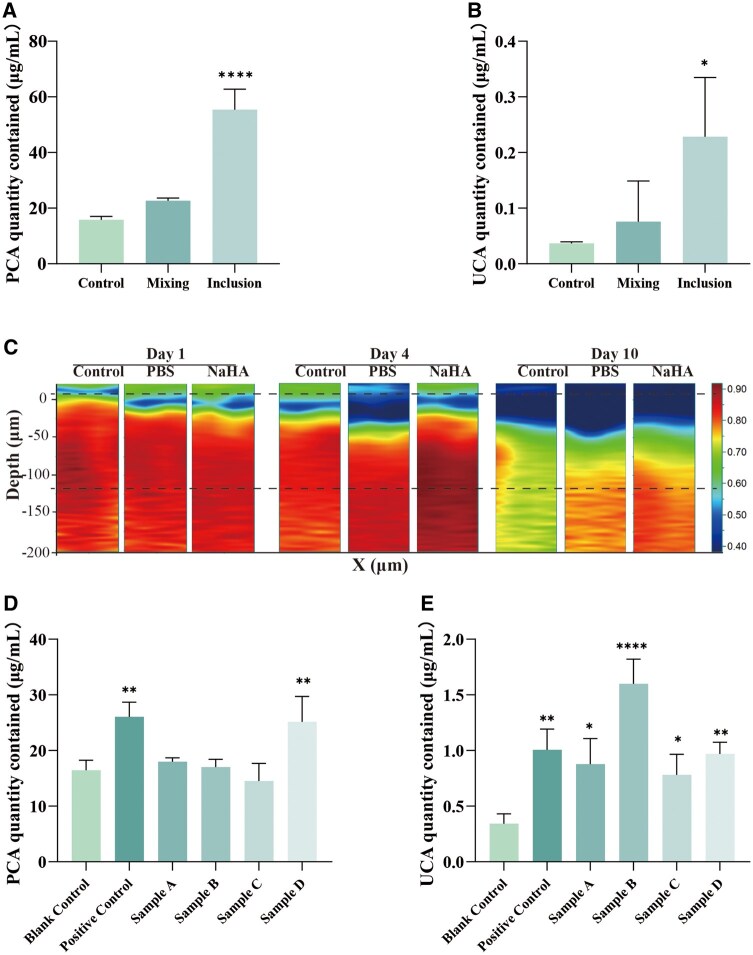
NaHA facilitates the preservation of a hydrated state in the RhFS system. Quantification of (**A**) PCA and (**B**) UCA levels in RhFS models, treated with mixing or inclusion strategies (*n* = 3). *P < 0.05, ****P < 0.0001, compared to the control. (**C**) Raman imaging of water molecules across the skin depth in the RhFS model on 1, 4 and 10 days of the air–liquid interface culture. The dashed lines mark the approximate epidermal region (∼0–120 μm) selected for hydration analysis. Comparative analysis of (**D**) PCA and (**E**) UCA levels in models treated with four different products (Sample A–D) via the inclusion method (*n* = 3). *P < 0.05, **P < 0.01, ****P < 0.0001, compared to the blank control.

To evaluate the moisturizing capacity, we quantified levels of key NMFs, PCA and UCA, in the epidermis layer of RhFS models. Consistent with the surface marker immunofluorescence staining results, the inclusion-based delivery strategy markedly increased the levels of NMF components, including PCA and UCA (Figure 7A and B). To further elucidate the impact of this delivery mode on tissue-level hydration dynamics, we subsequently applied the same strategy to deliver PBS or NaHA into the superficial dermis and performed confocal Raman imaging to monitor time-dependent changes in tissue water content at the epidermal–dermal interface.

At Day 1, no appreciable differences in Raman water signals were observed among the control, PBS and NaHA groups. In contrast, by Day 4 and Day 10, the NaHA-treated group exhibited substantially stronger water-related Raman signals than the PBS group, indicating a sustained hydration response within the epidermal compartment (Figure 7C). These results suggest that the superior bound-water capacity of NaHA enables prolonged maintenance of epidermal water accumulation. Moreover, the concomitant upregulation of NMFs likely acts synergistically to enhance the skin’s water-retention capacity, thereby reinforcing and extending the hydration effect at the tissue level.

Finally, we applied the RhFS model combined with the novel inclusion-based delivery strategy to evaluate the levels of NMFs following treatment with four different samples. The results demonstrated that only Sample D was capable of simultaneously upregulating both PCA and UCA (Figure 7D and E). Notably, this outcome was consistent with our previous assessments of the water-binding capacity of the corresponding product matrices, thereby supporting the distinguishing capability of the proposed evaluation framework.

## Discussion

### Establishment of a human-relevant evaluation framework

The rapid expansion of NaHA-based injectable hydrating products has revealed a clear gap in preclinical efficacy evaluation. Currently, product performance is assessed predominantly through clinical studies, which are constrained by subjectivity, inter-individual variability, limited mechanistic resolution, and high costs [20–22]. Conventional animal models exhibit limited translational relevance for injectable hydrogels, due to anatomical differences in skin structure and inadequate material retention following injection [23]. Furthermore, large-animal models are impractical for routine testing and mechanistic investigations [24]. Collectively, these limitations underscore the need for human-relevant, reproducible and mechanism-informed *in vitro* evaluation strategies for injectable hydrating biomaterials.

To address these challenges, this study establishes an integrated *in vitro* evaluation framework centered on a human stem cell-derived RhFS. By combining physicochemical water-binding properties of NaHA with cellular responses under dehydration stress and tissue-level functional outcomes, this framework provides a coherent assessment pathway spanning material, cellular and tissue scales. Importantly, the proposed strategy emphasizes clinically relevant intradermal delivery conditions, rather than relying on homogeneous exposure paradigms, thereby improving the relevance of *in vitro* evaluation for injectable products.

### Mechanistic insights: the dermal–epidermal hydration cascade

**Figure 8:**
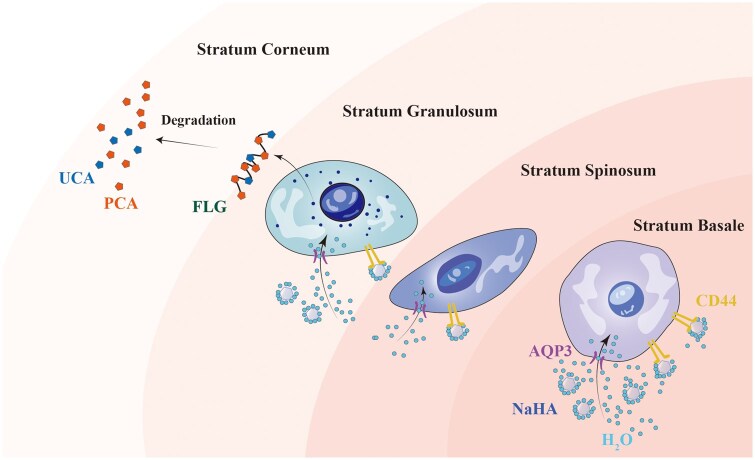
Schematic illustration of the moisturizing cascade induced by NaHA in the RhFS model.

Based on spatial and tissue-level considerations, the moisturizing performance of NaHA can be interpreted through a hierarchical dermis-to-epidermis process. Following intradermal placement in the superficial dermis, NaHA contributes to a local microenvironment characterized by enhanced water association, consistent with its intrinsic water-binding capacity. This hydration-favorable microenvironment supports sustained epidermal hydration responses at the dermal–epidermal interface (Figure 8).

As NaHA and associated water diffuse toward the epidermis, keratinocytes respond through enhanced interaction with hyaluronate via the CD44 receptor, promoting pericellular water retention [18]. The resulting increase in local hydration facilitates AQP3-mediated water transport into epidermal cells, contributing to intracellular hydration and epidermal water homeostasis [25]. At the upper epidermal layers, improved hydration supports FLG metabolism, and subsequent degradation in the SC generates NMFs, including PCA and UCA, which further enhance water retention and barrier function [26]. Together, these observations support a coherent mechanistic rationale linking dermal NaHA localization with epidermal moisturization, and provide a biological basis for the selection of evaluation markers used in this study.

### Importance of delivery mode: the ‘depot effect’ of the inclusion strategy

A key finding of this work is that the mode of NaHA delivery critically influences the detectability of moisturizing performance *in vitro*. Compared with homogeneous mixing, the inclusion-based delivery strategy consistently resulted in stronger hydration-related responses at molecular and functional levels. This difference is attributed to the ability of the inclusion method to place NaHA at a defined dermal position, thereby better approximating the clinical ‘depot effect’ following intradermal injection.

Localized placement of NaHA is expected to establish sustained hydration gradients at the dermal–epidermal junction [27], enhancing dermal–epidermal coupling and prolonging stimulation of basal keratinocytes [28]. These findings highlight that predictive *in vitro* models for injectable biomaterials must replicate not only the biological microenvironment but also key physical aspects of clinical delivery, including spatially controlled delivery and material retention.

### Structure–activity relationship: the role of bound water

The sensitivity of the proposed platform was further demonstrated by its ability to differentiate the performance of four commercial NaHA formulations (Samples A–D). Notably, Sample D exhibited the most significant efficacy in promoting PCA and UCA generation.

To understand the physicochemical basis of this superior performance, we revisited the Raman spectroscopy data. While all samples possessed similar chemical identities, Sample D was distinct in its water-binding state. Deconvolution analysis revealed that Sample D contained the highest proportion of ‘totally bound water’ (16.6%) compared to the others (2.6–5.3%).

We hypothesize that this specific state of water, tightly associated with the polymer chains, is less prone to rapid diffusion and evaporation. This property potentially prolongs the retention of the ‘hydration depot’ within the RhFS model, extending the duration of CD44 receptor activation. This correlation suggests a vital structure-activity relationship: the microscopic water-binding architecture of NaHA [29], rather than just concentration or average molecular weight, acts as an important determinant of its macroscopic moisturizing efficacy.

### Implications for quality control and regulatory science

From a regulatory perspective, this platform offers a substantial advancement over traditional methods. Existing animal models often fail to capture the subtle functional differences between formulations due to species-specific skin physiology. In contrast, our human-relevant platform successfully distinguished between commercial products that appeared similar in basic physicochemical tests. This sensitivity makes the RhFS-inclusion platform a valuable tool for other products [30]. It also can serve as a biological potency assay to complement routine physicochemical testing. Furthermore, by replacing animal testing, this approach aligns with the global regulatory trend toward the 3R principles, providing an ethically sound strategy for the standardization of injectable aesthetic products.

### Limitations and future directions

Despite these promising results, our study has limitations. The RhFS model lacks vasculature and immune components, preventing the assessment of systemic absorption or inflammatory responses. Additionally, this study focused on pure NaHA; future work should explore formulations with composite active ingredients. Nevertheless, by standardizing the inclusion-based administration technique, we present a reproducible, mechanistically informative and scalable system that bridges the gap between laboratory characterization and clinical efficacy for injectable biomaterials.

## Conclusion

Driven by the limitations of current approaches for evaluating injectable NaHA, this study establishes an integrated *in vitro* evaluation system as a human-relevant alternative to animal testing. By combining cellular assays with a full-thickness 3D skin model, the proposed framework enables reproducible assessment of hydration-related effects under clinically relevant delivery conditions. This system addresses key translational shortcomings of conventional animal models and provides a practical tool for preclinical performance evaluation of injectable hydrating biomaterials. Future efforts will focus on standardizing administration procedures and refining evaluation protocols to support broader application in product development and regulatory assessment.
